# Increased Grey Matter Associated with Long-Term Sahaja Yoga Meditation: A Voxel-Based Morphometry Study

**DOI:** 10.1371/journal.pone.0150757

**Published:** 2016-03-03

**Authors:** Sergio Elías Hernández, José Suero, Alfonso Barros, José Luis González-Mora, Katya Rubia

**Affiliations:** 1 Department of Ingeniería Industrial, Universidad de La Laguna, Tenerife, Spain; 2 Centro de Salud Jazmín, Sermas, Madrid, Spain; 3 Department of Psychology, Universitat Jaume I, Castellón, Spain; 4 Department of Fisiología, Universidad de La Laguna, Tenerife, Spain; 5 Institute of Psychiatry, Psychology and Neuroscience, King’s College London, London, United Kingdom; Centre de Neuroscience Cognitive, FRANCE

## Abstract

**Objectives:**

To investigate regional differences in grey matter volume associated with the practice of Sahaja Yoga Meditation.

**Design:**

Twenty three experienced practitioners of Sahaja Yoga Meditation and twenty three non-meditators matched on age, gender and education level, were scanned using structural Magnetic Resonance Imaging and their grey matter volume were compared using Voxel-Based Morphometry.

**Results:**

Grey matter volume was larger in meditators relative to non-meditators across the whole brain. In addition, grey matter volume was larger in several predominantly right hemispheric regions: in insula, ventromedial orbitofrontal cortex, inferior temporal and parietal cortices as well as in left ventrolateral prefrontal cortex and left insula. No areas with larger grey matter volume were found in non-meditators relative to meditators.

**Conclusions:**

The study shows that long-term practice of Sahaja Yoga Meditation is associated with larger grey matter volume overall, and with regional enlargement in several right hemispheric cortical and subcortical brain regions that are associated with sustained attention, self-control, compassion and interoceptive perception. The increased grey matter volume in these attention and self-control mediating regions suggests use-dependent enlargement with regular practice of this meditation.

## Introduction

The distribution of Grey Matter Volume (GMV) in our brain has an important role, among others on our mental health, our behaviour and our cognitive functions. In normal development, GMV and cortical thickness increase in childhood, peak in adolescence and then decrease progressively with age across adulthood, due to a combination of progressive and regressive changes such as synaptic pruning, white matter development and neuronal loss in older age [1, 2]. There is considerable evidence of neuronal plasticity, which is higher in childhood but still considerable in adulthood with evidence that neuronal interconnections are modifiable networks that change in their structure as a consequence of behavioural or cognitive training or skill learning [3]. Thus, a close interrelationship has been demonstrated between localized use-dependent enlargement of GMV, of neuronal activation and the corresponding enhancement of functions or capabilities [4, 5]. Inversely, there is consistent evidence that abnormalities in GMV are associated with a reduction of cognitive and behavioural functions, reinforced by the fact that regional GMV is typically abnormal in mental disorders [6–8].

Research of meditation has experienced an important growth over the last two decades, among other things due to evidence for beneficial effects on mental and physical health and for therapeutic benefits for a range of mental and psychosomatic disorders [8–12].

Meditation has been associated with differences in brain structure both in cross-sectional imaging studies that compared long-term meditators with non-meditators and in short-term longitudinal imaging studies that assessed brain structure changes in novices after weeks of meditation practice (for review, see [13]). While longitudinal imaging studies can allow for more causal inferences, the cross-sectional comparison of long-term meditators with non-meditators can shed more light on the longer-term effects of meditation.

Several brain areas have consistently been shown to be different in several morphometry studies of long-term meditation [13], as well as been shown to be activated in fMRI studies of meditation, including the insular cortex [14–17], ventrolateral prefrontal cortex (VLPFC) [14, 18], somatomotor cortices [14, 19], inferior temporal gyrus [15, 18, 20, 21], fusiform gyrus [17, 18, 22], rostrolateral prefrontal cortex [14, 18, 22], anterior cingulate cortex [18, 23, 24] and hippocampus [25, 26]. Most of these areas mediate the top-down control or processing of attention or emotions, which is also the target of most meditation techniques [8, 13].

However, there is large heterogeneity among meditation techniques, which often involve very different practices and which are likely to have different effects on the brain. So far, the following meditation techniques have been studied using brain structure imaging techniques: Zen meditation [23, 27], Tibetan Buddhist meditation [22], Mindfulness meditation [25, 28, 29], Soham [30], Loving-kindness meditation [21], Brain wave vibration [18], or a combination of different meditation techniques within the same study: Chenrezig—Kriya—Shamatha—Vajrayana—Vipassana and Zazen [17, 20, 26, 31–33].

Meditation, as originally conceived in in the East by Patanjali [34, 35], is aimed to reduce thoughts to ultimately reach the state of thoughtless awareness which is considered a different state of consciousness where one is fully perceptually alert, yet has no thoughts. It is consequently described as a state of pure attention without any thought content [8]. This state of pure “contentless attention” appears to be subjectively associated with feelings of positive emotions and is described in Sanskrit as the state of “Sat Chit Ananda” which translates as a state of pure attention and joy [8, 36]. Sahaja Yoga Meditation (SYM) shares some goals with some other meditation techniques such as Mindfulness meditation, Loving-kindness meditation or other Buddhist meditations i.e., to be fully conscious on the present moment, to reduce the wandering mind and to increase compassion and love. However, one of the distinctive features of SYM is that the practitioner experiences the state of mental silence or “thoughtless awareness” on a regular basis in their meditation (if not necessarily daily), which makes it suitable to test the neural correlates of this unique state of thoughtless awareness which is the ultimate goal of traditional meditation, as originally conceived in the East [35].

SYM has been shown to have beneficial effects on a range of physical, mental and neurological disorders including: asthma [37], high blood pressure [38], menopause [39], epilepsy [40–42], depression [43], anxiety [38], work stress [44], and Attention-Deficit/Hyperactivity Disorder [45].

Several studies have used electroencephalography (EEG) to investigate the effects of SYM on brain activation. These studies showed increased theta and alpha activity over fronto-central parietal brain regions in long-term meditators compared to short-term meditators of SYM, which furthermore was associated with the state of mental silence. Furthermore, increase of theta activity over left frontal and limbic regions was associated with the feelings of joy associated with the state of mental silence. Fronto-parietal activation has been associated with sustained attention [46] while increased frontal alpha activity is thought to reflect a reduction in brain regions that mediate mental effort and external attention [47]. A more recent evoked potential study [48] showed that long-term SYM practice in long-term meditators compared to non-meditators was associated with increased efficiency of attention-related activity over predominantly right hemispheric fronto-central regions during the processing of salient emotional stimuli, which the authors interpreted as increased top-down control over fast automatic salience detection, presumably based on amygdala function. The findings reflect enhanced frontal lobe mediated top-down attention control and emotion regulation in long-term practitioners of SYM.

Only one previous study, from our group, used fMRI to assess brain activation during the meditation in the MRI scanner in long-term practitioners of SYM [49]. The study showed that, during their meditation, long-term SYM practitioners appear to pass through an initial intense neural self-control process necessary to silence their mind with neural activity in bilateral VLPFC/ insula and temporal regions. After this initial process, meditators experienced a relatively reduced brain activation concomitant with the deepening of the state of mental silence with a remaining activity in right rVLPFC/ insula and right middle/superior temporal cortex, with the activation in rVLPFC/insula being directly linked to the subjective depth of the meditation reported by the meditators. We interpreted the activity over rVLPFC as a reflection of the state of enhanced sustained attention during the state of mental silence.

In conclusion, functional imaging studies of long-term SYM practice report activation during meditation in fronto-parieto-temporal regions of sustained attention and in limbic regions of affect control [48–50]. However, nothing is known on the long-term effects of SYM on brain morphometry.

The aim of this study was therefore to test whether the acute activation differences we observed in fMRI during meditation relative to rest in long-term practitioners of SYM would also be reflected in regional grey matter differences on brain structure in long-term SYM practitioners relative to non-meditators, due to use-dependent plasticity. For this purpose, we compared GMV between a group of experienced SYM practitioners and a group of non-meditators, matched on age, education status and other demographic variables. Based on our fMRI and previous EEG findings on SYM [48–51], and based on the evidence for grey matter morphometry changes in long-term meditators of other meditation traditions in areas of affect and attention control [13], we hypothesised that experienced practitioners of SYM would have larger GMV in regions mediating attention and affect control, including: insula, VLPFC, ACC, the parieto-temporal junction, and temporo-limbic regions.

## Materials and Methods

### Participants

Forty six right-handed, white Caucasian, healthy volunteers, between 21 and 63 years, participated. Twenty three experts in SYM (17 females) were compared with 23 non-meditators (17 females). Groups were matched on age, gender, education degree and body mass index (see Table 1). Volunteers had no physical or mental illness, no history of neurological disorders, and no addiction to nicotine, alcohol or drugs.

**Table 1:** Demographic characteristics of the group.

	Meditators Mean (SD)	Controls Mean (SD)	t(df = 44)	p-value*
Volunteers N°	23	23		
Age (years)	46.5 (11.4)	46.9 (10.9)	-0.13	0.89
Age range (years)	20.3–63.1	21.3–63.3		
Education degree, 0 to 6	3.78 (1.2)	4.04 (1.36)	0.69	0.50
Height (cm)	167.0 (8.8)	167.2 (7.6)	0.09	0.93
Weight (Kg)	69.5 (14.6)	71.7 (14.5)	0.53	0.60
Body mass index	24.9 (4.5)	25.5 (3.9)	0.54	0.60

* p-values represent group differences between meditators and controls using two-tailed independent samples t-tests.

Meditators were recruited from the local Tenerife SYM group in addition to SYM practitioners attending a seminar of Sahaja Yoga in Tenerife in January 2014. Controls were recruited through local and facebook advertisements. Controls did not practice any type of meditation or yoga. Only 3 controls reported a minimum meditation experience of less than 6 months practice.

Meditators had between 5 and 26 years of experience of daily meditation practice in SYM (mean:14.1 SD (6.1) years) and the average time dedicated daily to meditation per day was 84.7 (32.2) minutes.

All participants filled in different questionnaires to evaluate their individual health status, education and age. Meditators additionally were given a questionnaire to register their experience in SYM such as: years of practice, total hours of meditation, average time dedicated to meditation per day and frequency of the perception of the state of mental silence.

All participants signed informed consent to participate freely. This study was approved by the Ethics Committee of the University of La Laguna.

### MRI Acquisition

All images were obtained on a 3T MRI Scanner, using an echo-planar-imaging gradient-echo sequence and an 8 channel head coil. A high-resolution T1-weighted three-dimensional inversion recovery spoiled gradient echo sequence was used to image the whole brain and the brainstem. A 3D fast spoiled-gradient-recalled pulse sequence was obtained with the following parameters: TR = 8.761 ms, TE = 1.736 ms, flip angle = 12°, matrix size = 256 x 256 pixels, spacing between slices and slice thickness = 1 mm, voxel resolution = 0.98 x 0.98 x 1 mm. Total acquisition time was 13 minutes.

### Voxel-Based Morphometry

Voxel-based morphometry (VBM) [52] with DARTEL was conducted using SPM12 software package (Statistical Parametric Mapping software: http://www.fil.ion.ucl.ac.uk/spm/). Processing steps were performed as suggested by Ashburner [53]. VBM with DARTEL has been shown to be more sensitive than standard VBM [54] and provides results comparable to those achieved with manual segmentation [55].

The procedure followed these steps: 1. All T1-weighted anatomical images were displayed to screen to verify they were free from gross anatomical abnormalities. 2. For better registration, the T1 images were manually centred at the anterior commissure and reoriented according to the anterior–posterior commissure line. 3. Images were segmented into: Grey matter (GM), White matter (WM) and Cerebrum Spinal Fluid (CSF), using the New Segment procedure in SPM12, a segmentation that provides acceptable substitute for labour intensive manual estimates [56]. 4. The DARTEL routine inside SPM12 was used to spatially normalize the segmented images [54]. The image intensity of each voxel was modulated by the Jacobian determinants to ensure that regional differences in the total amount of GMV were conserved. 5. The registered images were then transformed to Montreal Neurological Institute (MNI) space using affine spatial normalization. 6. Finally, the normalized modulated GMV images were smoothed with a 4-mm full-width at half-maximum (FWHM) isotropic Gaussian kernel to increase the signal to noise ratio.

For each individual, total GM, WM and CSF were obtained with the Matlab script ‘get_totals.m’ [57] and used to calculate the individual Total Intracranial Volume (TIV) by summing the volumes of the three already mentioned components (GM, WM, CSF).

### Statistical Analysis

Differences in GMV between meditators and controls were analysed by conducting an ANCOVA analysis in SPM12. In order to control for inherent differences in brain structure, TIV, age, and gender were included as nuisance covariates [53]. An absolute threshold masking of 0.1 was used to avoid edge effects around the borders between GM and WM, which meant that voxels with a GM probability below 0.1 were removed from the analyses [53].

Because structural images display local variation in smoothness, cluster level correction was applied using random field theory and non-stationary correction [58]. Statistical thresholds were set at corrected p-values determined by non-stationary cluster-level correction with family wise error p(FWE-corr)<0.05 with uncorrected voxel-level of p<0.001.

To determine whether GMV clusters that differed between groups were correlated with the subjective measures of the meditation experience within the meditation group, a second-level SPM regression analysis was used to correlate these GMV clusters within the meditator group with the number of years of their meditation practice, the total hours meditated overall, the average time per day dedicated to meditation and the frequency of the perception of the state of mental silence. TIV, age, and gender were also included here as nuisance covariates.

## Results

### Demographic characteristics of the groups

As shown in Table 1, the meditation and non-meditation groups did not differ in any demographic measures such as mean age, age range, height, weight, education status or body mass index.

The meditation group had been meditating between 5 and 26 years, with an average (standard deviation) of 14 (6) years. Their total hours of meditation were between 1213 to 12558 hours, with a mean of 5773 (3401) hours. Their daily practice was between 34 and 150 minutes, amounting to an average of 84 (32) minutes.

### TIV and GMV analysis

ANCOVA showed that TIV did not significantly differ between groups (F = 1.070, p = 0.307), using gender and age as nuisance covariates, meditators: TIV mean (SD) = 1385.3 mL (157.7), non-meditators: 1350.5 mL (172.4). In all other statistical analyses, TIV, gender and age were always entered as nuisance covariates.

**Fig 1:**
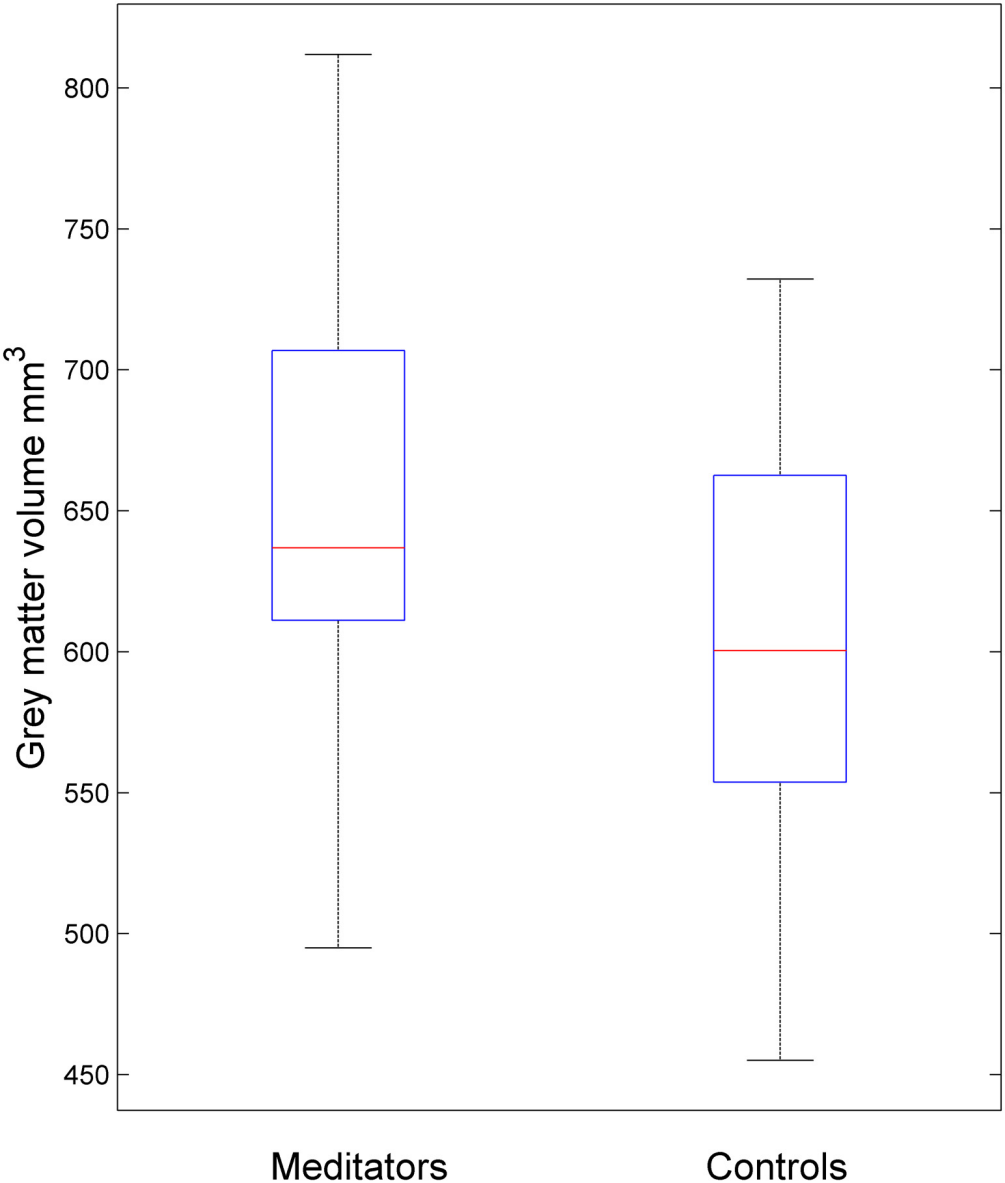
Box plot of grey matter volumes across the whole brain of meditators and controls. The central box represents the value from the lower to upper quartile (25th to 75th percentile). The middle line represents the median. The vertical line extends from the minimum to the maximum value.

ANCOVA with TIV, gender and age as nuisance covariates showed that overall whole brain GMV was significant different between groups (F = 10.445, p = 0.002), due to GMV being larger in meditators than non-meditators, meditators GMV mean (SD) = 653.4 mL (87.0); non-meditators 611.0 mL (74.6), (see Fig 1).

**Fig 2:**
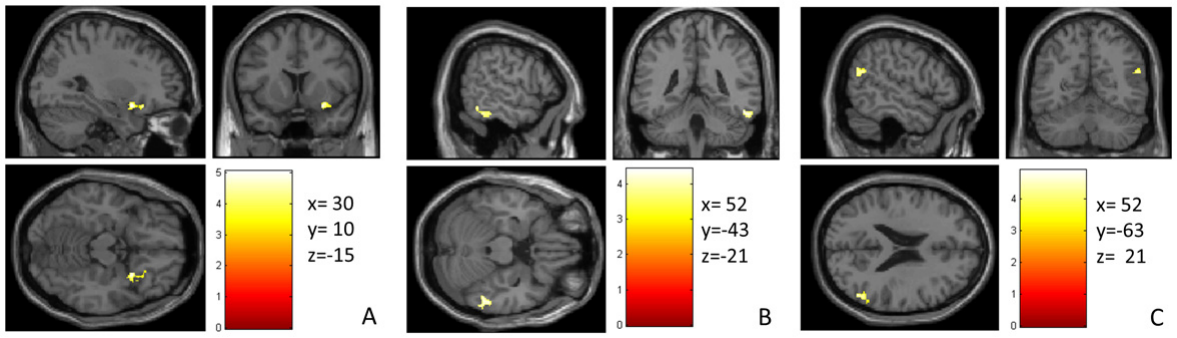
Areas where meditators exhibited larger GMV than non-meditators. (A) right insula/vmOFC, (B) right inferior temporal gyrus and (C) right angular gyrus. The color intensity represents *T*-statistic values at the voxel level.

Regional analyses showed that two brain regions were significantly larger in GMV in meditators compared to non-meditators (p (corrected) <0.05): the right anterior insula, extending rostrally into right ventromedial orbitofrontal cortex (vmOFC) and the right inferior temporal gyrus extended to the right fusiform gyrus. There was also a trend-finding for the right inferior parietal lobe (right angular gyrus), which was observed at a p-corrected cluster-level p = 0.069, (see Table 2 and Fig 2).

**Table 2:** Brain regions where meditators had significantly larger GMV than non-meditators.

Region	Side	Brodmann Area	Cluster size mm^3^	x	y	z	T of peak	Cluster p-value*
Insula, vmOFC	R	13, 47	564	30	10	-15	5,02	0,023
Inf. Temporal Gyrus, Fusiform Gyrus	R	20,37	739	52	-43	-21	4,43	0,037
Angular Gyrus**	R	39	476	52	-63	21	4,87	0,069**

x, y, z, (mm) MNI co-ordinates of peak,

* the corrected p-values were determined by non-stationary cluster-level correction based on family wise error p(FWE-corr)<0.05 with uncorrected voxel-level p<0.001, R = right side, Side = side of the hemisphere. vmOFC = ventromedial orbitofrontal cortex,

** only observed at a trend-level of p(FWE-corr) = 0.069.

**Fig 3:**
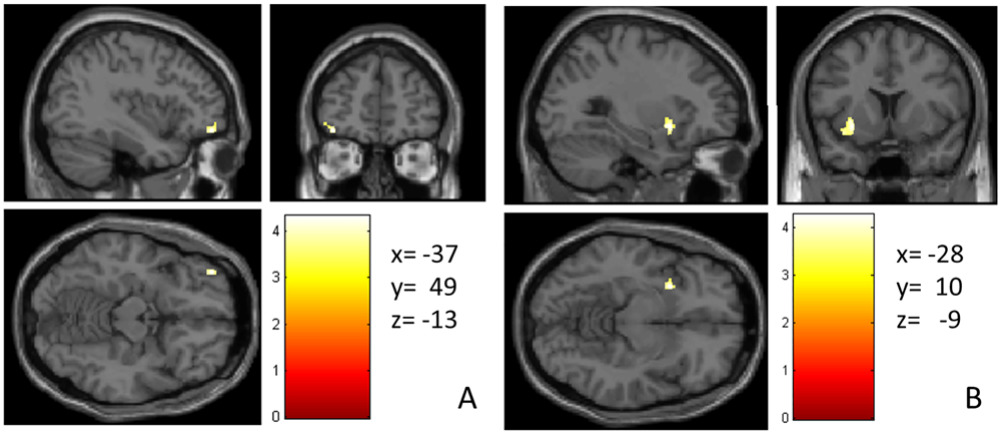
A priori hypothesised regions that showed larger GMV in meditators relative to controls at a more lenient threshold. (A) left VLPFC (B) left anterior insula. The color intensity represents *T*-statistic values at the voxel level.

Given that in our previous fMRI study we found that meditation elicits most prominently activation in VLPFC and insula [49], we wanted to test our a priori hypothesis that these regions would also be larger in the VBM analysis due to use-dependent plasticity. Therefore, to test our hypothesis of enhanced structure in VLPFC and insula, the data were re-analysed with a more lenient voxel-wise threshold of p < 0.005 and non-stationary cluster-level false discovery rate p(FDR-corr) p < 0.05. In fact, as expected, a cluster in left VLPFC (peak voxel MNI coordinates x, y, z: -37, 49, -13: cluster p-corr = 0.04) and a cluster in left anterior insula (peak voxel MNI coordinates x, y, z: -28, 10, -9, cluster p-corr = 0.04) were larger in meditators relative to non-meditators, (see Fig 3). There were no regions where non-meditators had larger GMV than mediators.

### Correlation analyses

There were no significant correlations within the meditation group between the total GMV or the GMV in any of the regions that differed between groups and the meditators’ experience, including number of years of their meditation practice, the total hours meditated overall, the average time per day dedicated to meditation or the frequency of the perception of the state of mental silence.

## Discussion

To our knowledge, this is the first study that tested the association between long-term SYM, which elicits the state of mental silence on a regular basis, and brain morphometry. Long-term practitioners of SYM showed larger GMV in predominantly right hemispheric brain areas such as the ventromedial orbitofrontal cortex (vmOFC), the insula, and the inferior temporal lobe. These areas are associated with affect and attention control, interoception and autonomic function surveillance. At a more lenient level, we also observed enhanced GMV in left VLPFC and left insula. The findings extend our previous fMRI results by showing that attention and limbic regions that are activated during the practice of meditation in long-term meditators such as VLPFC/insula and temporal lobe are also larger in structure in long-term meditators, presumably due to use-dependent plastic effects of years of SYM practice on these brain structures.

The findings of larger right and left insula in practitioners of SYM relative to non-meditators are in line with the effects observed in other meditation techniques. The anterior insular cortex is one of the most replicated areas that have been shown to be enlarged in structure in studies of meditation, most commonly in the right hemisphere [14, 15, 18], but also bilaterally as in this study [17]. The insula is associated with interoception, visceral and emotional self-awareness, including awareness of respiration and heart rate [59]. The anterior insula as well as inferior temporal regions are also part of the cingular-opercular salience detection network and the insula has been shown to mediate tonic and intrinsic alertness [60], which may reflect the subjective experience during meditation of enhanced alertness and attention during the state of mental silence [61]. This difference between meditators and controls presumably reflects the inner attention training during SYM that is common with other types of meditation like Vipassana or Mindfulness Meditation [62, 63] and which may be associated with enhanced body-self-awareness and increased intrinsic self-awareness and alertness during the meditation [13, 60].

This larger GMV in the insula may also play a role in the emotional and clinical benefits shown to be engendered by meditation [9, 12], given that larger GMV in bilateral insula has been associated with “good life”, traits concerned with personal growth, self-acceptance, purpose in life and autonomy (among others), and been shown to be a good predictor of good health [64]. Right insula GMV increase has also been associated with improvements to identify and describe emotions in the self after a brief 8 weeks- Mindfulness Based Stress Reduction training program [65]. Emotional intelligence has also been shown to correlate positively with larger GMV in left insula and rVLPFC in a study in 328 university students [66].

The right insula cluster extended to the vmOFC that is well connected with sensory regions and part of the limbic affect regulation system, exerting top-down control over amygdala, ventral striatum and hypothalamus [67]. While amygdala and striatum store and generate a more automatic reaction to stimuli based on past experiences, the vmOFC generates a more flexible assessment to counterbalance the automatic reactivity in amygdala and striatum and has hence an important role in top-down emotional regulation and reappraisal of negative emotional states [48, 68, 69].

The enlarged GMV in this vmOFC affect control region may be related to the subjective experience of the state of mental silence, which is associated with feelings of positive emotions and a detached “witness state”. In EEG studies, the subjective feelings of happiness during SYM have been associated with activation over fronto-temporal regions [70]. Furthermore, long-term practitioners of SYM showed greater emotional stability and greater detachment than non-meditators during the exposure to stressful aversive film viewing, as shown in lower subjective emotional ratings of the response to the stressful stimuli, a reduction in parasympathetic stress indicators such as skin conductance measures, and reduced emotional arousal, indexed by alpha and gamma EEG oscillations [51]. Last, long-term SYM practitioners relative to non-meditators showed increased attention-related right hemispheric fronto-central activation during the processing of emotionally salient stimuli, suggesting increased frontal top-down control over automatic saliency processing regions and hence enhanced cognitive and affective control [48]. This enhanced affect control, in particular over negative emotions, is likely to be mediated by vmOFC top-down control over limbic brain regions. The enlarged GMV in vmOFC could also potentially be associated with findings that several weeks of SYM treatment has been shown to reduce symptoms of depression and anxiety, which one could speculate may reflect an effect on ventromedial fronto-limbic brain systems of affect regulation [38, 43, 44].

We found a more dorsal cluster of rVLPFC and right insula to be activated in fMRI in long-term SYM mediators during their meditation in the scanner which was furthermore directly associated with the subjective depth of the state of mental silence [49]. Although the peak of the frontal cluster was in a more dorsal location, some of the cluster extent overlapped with the here observed ventromedial prefrontal location. Right fronto-parieto-temporal activation was also correlated with the state of mental silence in an EEG study of SYM [47, 48]. We hypothesized that the more dorsal location in rVLPFC that was associated with the “acute meditation” was related to the inhibitory self-control process needed to reject unwanted thoughts or distractions, which is crucial to reach and sustain the state of mental silence [49] and which is typically mediated by rVLPFC [71, 72]. Ventrolateral prefrontal cortex, anterior insula, inferior parietal and inferior temporal lobe together play a crucial role in attention, as they form part of the ventral attention system [73–75]. The practice of meditation has been considered an effective attention training [8], illustrated by improved performance in cognitive attention tasks in long-term meditators [12, 63, 76, 77] and this may well have led to plastic changes in regions of the right hemispheric ventral attention system that mediates sustained attention.

The enlarged GMV in right inferior temporal lobe is intriguing. As mentioned above, the bilateral inferior posterior temporal lobes are part of a generic cognitive control network that is activated during higher level and attention demanding tasks, independent of the task in question, and thus part of the dorsal and ventral attention systems [78–80]. The regular practice of meditation, if considered an effective attention and cognitive self-control training, may lead to the enlargement of some parts of this generic attention network. Previous morphometry studies of meditation have found larger GMV in inferior temporal gyrus, albeit in the left hemisphere [15, 18, 20, 21]. The integrity of this region is also key to prosopagnosia, where the emotional familiarity of a face is no longer recognised [81, 82]. Alternatively, the right inferior temporal lobe has also been associated with socio-emotional competence. Thus, a recent study found that right inferior temporal GMV was positively associated with individual variations of gratitude so that individuals with larger GMV expressed more gratitude [83]. Similarly, the increased volume of this region was associated in healthy individuals with higher competence in interpreting the intentions of others [84]. Also, WM volume in a slightly more superior right posterior temporal region has been associated with autism spectrum traits in healthy participants [85]. It thus appears that the integrity of this region may be related to socio-emotional abilities.

The right angular gyrus cluster that was larger at a trend-level (p-corr = 0.69) in long-term SYM practitioners is also part of the right temporo-parietal junction that is not only a crucial part of posterior attention networks [73–75] but has also been related to empathy. Thus the temporo-parietal junction is activated when we try to understand the feelings and thought of others or show empathy for others [86]. These are important capacities for an enriched emotional and social life that facilitates the emotions of love and compassion [87]. Altruism has also been shown to be linearly correlated with GMV in the temporo-parietal junction in a VBM research [88].

Although we observed at a more lenient threshold activation in left insula and left VLPFC, the areas that showed most significantly larger GMV in long-term meditators were in the right hemisphere. The findings are in line with the “acute” changes we observed in long-term meditators of SYM during their meditation in our fMRI study, where initially bilateral VLPFC/insula and temporal regions were activated which then became more prominently right hemispheric with the progressive depth of meditation. Predominantly right-hemispheric fronto-temporal activation [49] and fronto-temporo-parietal activation were also associated with the depth of thoughtless awareness in EEG studies [47]. Attention systems are more prominently right-hemispheric [73] which would fit the notion that meditation is a powerful attention training that enhances the plasticity of attention and saliency processing in right hemispheric fronto-insular-parieto-temporal regions. Furthermore, the right hemisphere is also more closely connected to the limbic system [89, 90] and mediates interoceptive functions [59, 91].

In addition to larger GMV in these brain regions of interoceptive perception, attention and/or affect processing, we also found that long-term practitioners of SYM compared to non-meditators had overall significantly larger GMV across the whole brain. This overall larger GMV, not previously observed in other meditation techniques, may be related to the differences of SYM relative to other meditation practices, most prominently the frequent achievement of the state of mental silence which is subjectively experienced as an altered state of consciousness. Given that GMV decreases with age and this is associated with the normal aging process [2], one could potentially speculate that long-term meditation is associated with a delay of normal age-related decline in GMV and hence a younger brain structure pattern overall.

An important limitation of this study, common to all cross-sectional studies of brain morphometry, is that brain morphometry differences cannot be attributed solely to meditation practice. Although we matched groups by age, gender, education level, and body mass, we cannot exclude that differences in lifestyles, or other unmeasured factors that may have differed between groups previous to the practice of meditation, could have influenced the brain morphometry measures.

Causal inferences are further hampered by the fact that we did not find any correlations between the brain structure changes and meditation–related measures such as years or hours of meditation in the SYM group, time dedicated to meditation per day or the subjective depth of the meditation experience overall. It is possible that the lack of correlation suggests a ceiling effect that may precede the 5 years of minimum accumulated practice of meditation in this study. Previous morphometric studies of meditation have shown inconsistent associations between GMV and the meditators’ experience (mainly years practising meditation or total hours meditated) [13–15, 20]. Given the lack of correlations, we cannot exclude that brain changes in long-term meditators may be associated with some other mediating or moderating factors that differ between people who chose to meditate and those who don’t. Future longitudinal VBM studies of SYM may be more powerful to infer direct causal effects between meditation practice and brain structure changes.

## Conclusions

This is the first brain morphometry characterization of the long-term practice of SYM. We show that long-term practitioners of SYM compared with non-meditators have statistically significant larger GMV across the whole brain, not previously observed in practitioners of any other meditation technique. Furthermore, GMV differed in several predominantly right hemispheric regions: in insula, ventromedial orbitofrontal cortex, inferior temporal and parietal cortices as well as in left ventrolateral prefrontal cortex and left insula.

These areas have been shown to be associated with sustained attention/cognitive control, emotion control, interoceptive perception and feelings of compassion, suggesting that long-term SYM practice may potentially enhance the functions mediated by these regions and consequently lead to neuroplastic enlargements.
